# Supplementary material for: Renewable formate from sunlight, biomass and carbon dioxide in a photoelectrochemical cell
Source: Nat Commun. 2023 Feb 23;14:1013. doi: 10.1038/s41467-023-36726-3 (PMC9950059; doi:10.1038/s41467-023-36726-3)
Supplement: Supplementary file 1 — Supplementary Information [file 41467_2023_36726_MOESM1_ESM.pdf]

Supplementary Information

Renewable formate from sunlight, biomass and carbon dioxide in a photoelectrochemical cell

Yuyang Pan[1]†, Huiyan Zhang[1]†, Bowen Zhang[1], Feng Gong[1], Jianyong Feng[2], Huiting Huang[2], Srinivas Vanka[3], Ronglei Fan[4], Qi Cao[1], Mingrong Shen[4], Zhaosheng Li[2]*, Zhigang Zou[2], Rui Xiao[1], Sheng Chu[1]**

[1]*Key Laboratory of Energy Thermal Conversion and Control of Ministry of Education, School of Energy and Environment, Southeast University; Nanjing 210096, China*

[2]*Collaborative Innovation Center of Advanced Microstructures, National Laboratory of Solid State Microstructures, College of Engineering and Applied Sciences, Nanjing University; Nanjing 210093, China*

[3]*Department of Electrical and Computer Engineering, McGill University, 3480 University Street, Montreal, Quebec H3A 0E9, Canada*

[4]*School of Physical Science and Technology, Jiangsu Key Laboratory of Thin Films, Collaborative Innovation Center of Suzhou Nano Science and Technology, Soochow University; Suzhou 215006, China*

†These authors contributed equally to this work.

*Corresponding authors Email: hyzhang@seu.edu.cn (H. Zhang)

zsli@nju.edu.cn (Z. Li)

schu@seu.edu.cn (S. Chu)

Supplementary Information includes:

Supplementary Figures 1-35

Supplementary Tables 1-3

Supplementary Note 1

References 1-34

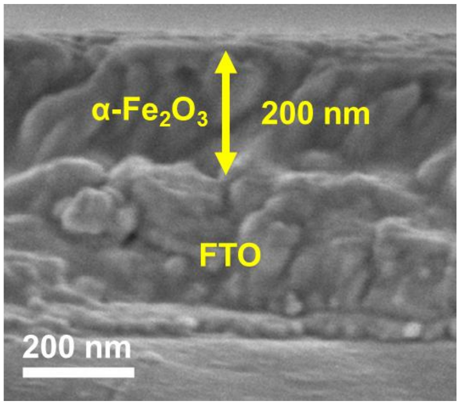

Supplementary Figure 1 | Side-view SEM image of α-Fe_2O_3 photoanode. It can be detected that α-Fe_2O_3 film with a thickness of 200 nm was formed over FTO glass.

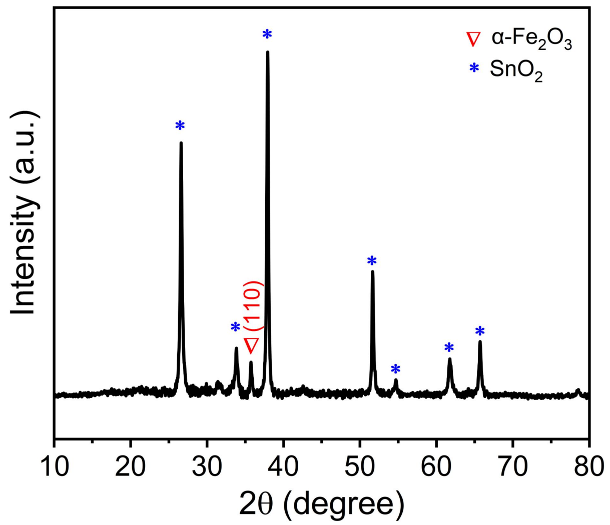

Supplementary Figure 2 | XRD pattern of α-Fe_2O_3 photoanode. XRD pattern reveals the pure hematite phase with predominant (110) facet, which has been widely considered as the preferred orientation with good conductivity and is beneficial for high PEC activity [1–4]. The other XRD peaks are ascribed to cassiterite SnO_2 phase from FTO substrate.

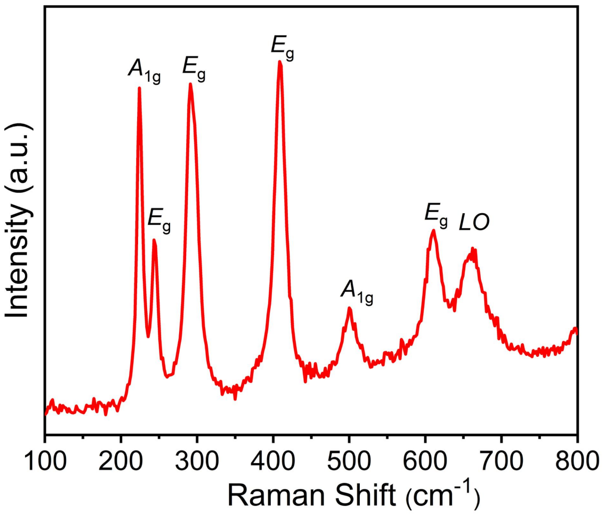

Supplementary Figure 3 | Raman spectrum of α-Fe_2O_3 photoanode. Raman spectrum exhibits the characteristic peaks of hematite phase with A_{1g} located at 223 and 494 cm^{-1} as well as E_g located at 243, 291, 408, and 606 cm^{-1} [5–7]. The peak at 660 cm^{-1} can be ascribed to the presence of forbidden longitudinal optical (LO) mode due to symmetry breakdown induced by structural disorders [8].

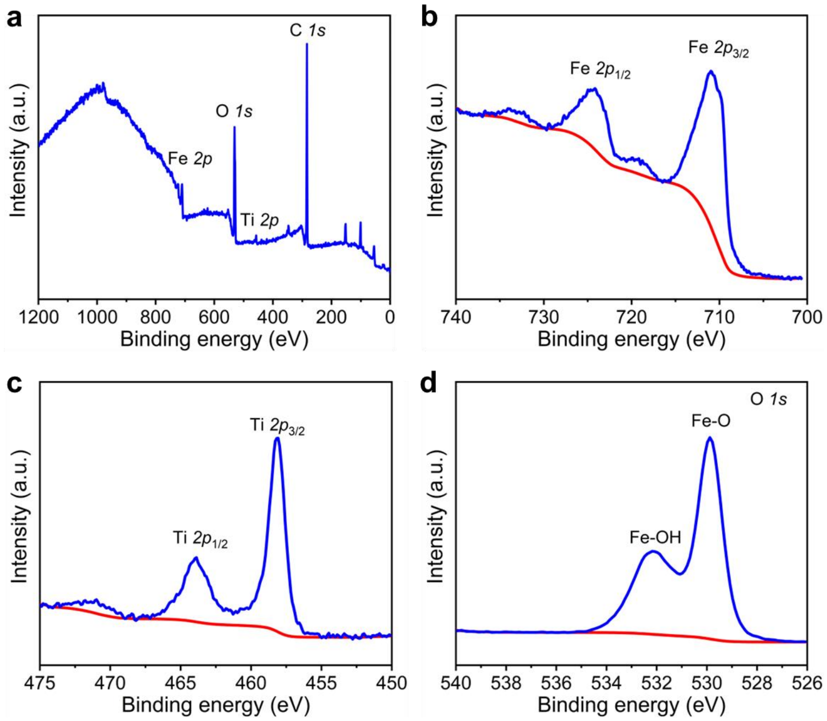

Supplementary Figure 4 | XPS spectra of α-Fe_2O_3 photoelectrode. (a) survey scan, (b) Fe *2p*, (c) Ti *2p* (d) O *1s*. All the spectral features are attributed to the constituent elements of α-Fe_2O_3 [9–12]. The α-Fe_2O_3 photoanode was doped with 3 at% Ti to enhance the electronic conductivity [13–15]. The strong Fe $2p_{1/2}$ and Fe $2p_{3/2}$ peak at 710.8 and 724.0 eV can be assigned to the Fe^{III} state from α-Fe_2O_3 [12]. A characteristic satellite peak of a-Fe_2O_3 also appeared at 718.8 eV [12]. The peaks located at 458.1 and 463.9 eV belong to Ti $2p_{3/2}$ and Ti $2p_{1/2}$, which correspond to Ti^{4+} in the Ti–O–Fe structure [9]. The O *1s* peaks located at 529.9 and 532.1 eV can be assigned to the lattice oxygen of α-Fe_2O_3, and hydroxyl groups of Fe–OH, respectively [12].

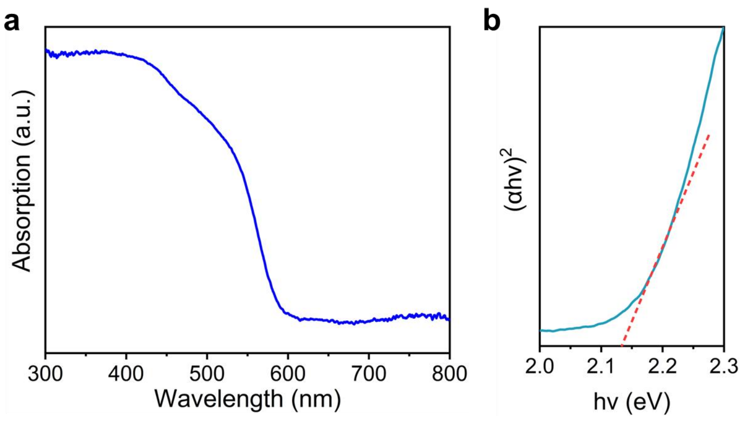

Supplementary Figure 5 | a UV–Vis spectrum and **b** tauc plot of α-Fe_2O_3 photoanode.

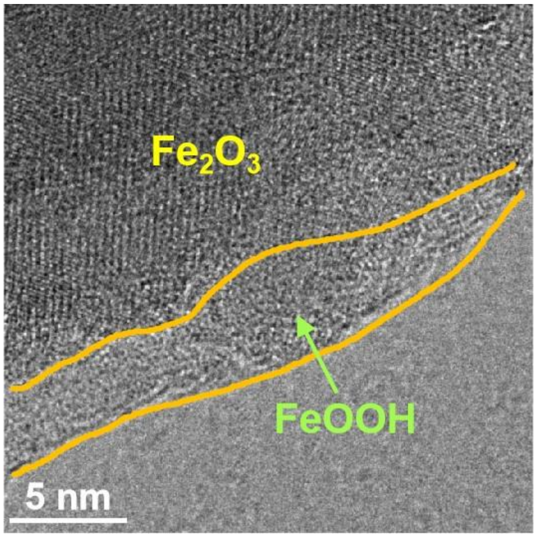

Supplementary Figure 6 | TEM image of FeOOH/α-Fe_2O_3.

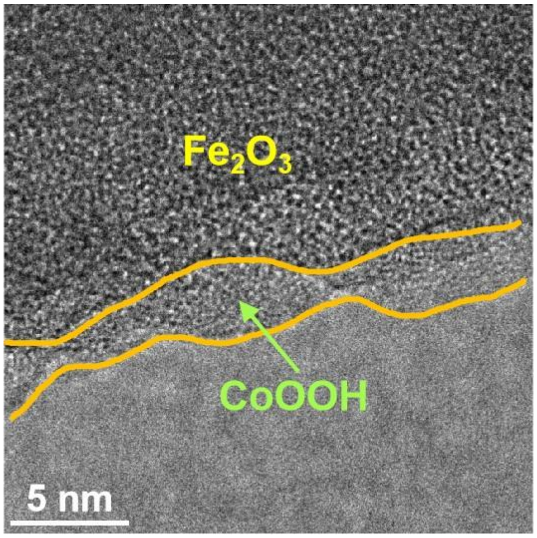

Supplementary Figure 7 | TEM image of CoOOH/α-Fe_2O_3.

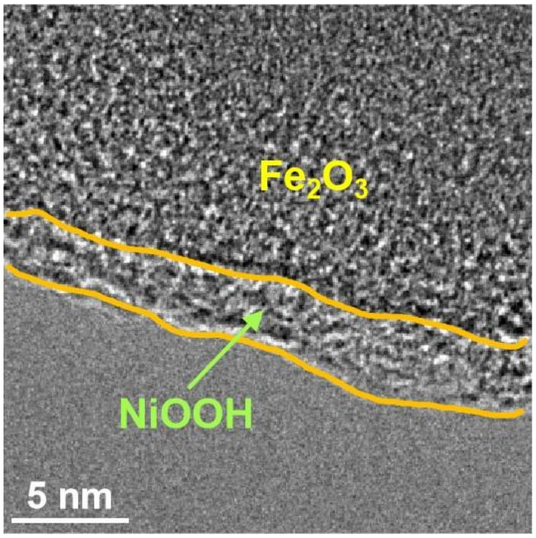

Supplementary Figure 8 | TEM image of NiOOH/α-Fe_2O_3.

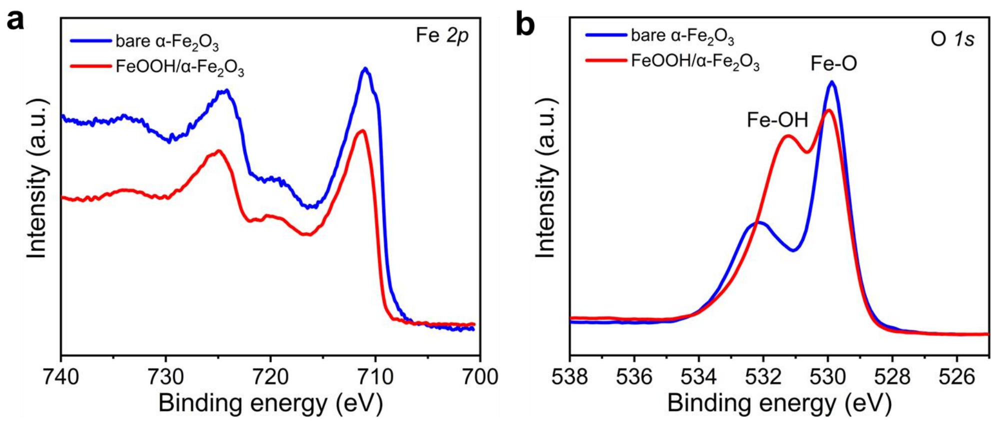

Supplementary Figure 9 | XPS spectra of (a) Fe *2p*, (b) O *1s* for bare α-Fe_2O_3 and FeOOH/α-Fe_2O_3. The O *1s* peak assigned to hydroxyl groups of Fe–OH had an obvious enhancement after the cocatalyst deposition, indicating the presence of FeOOH cocatalyst.

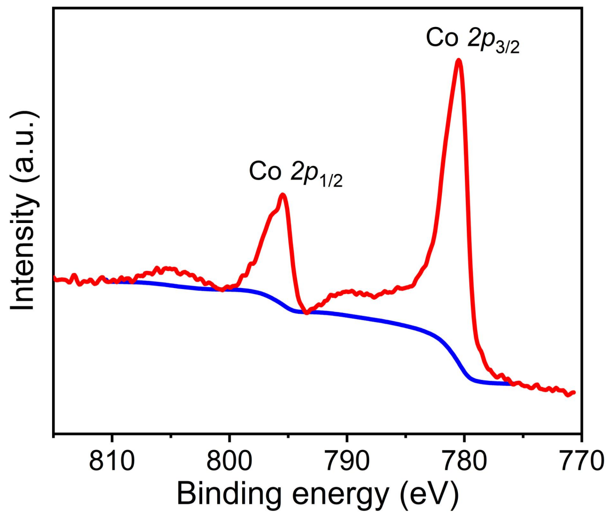

Supplementary Figure 10 | XPS spectrum of Co *2p* for CoOOH/α-Fe_2O_3. The Co $2p_{3/2}$ binding energy at 780.2 eV and Co $2p_{1/2}$ binding energy at 795.1 eV are assigned to Co^{III} state from CoOOH [16,17].

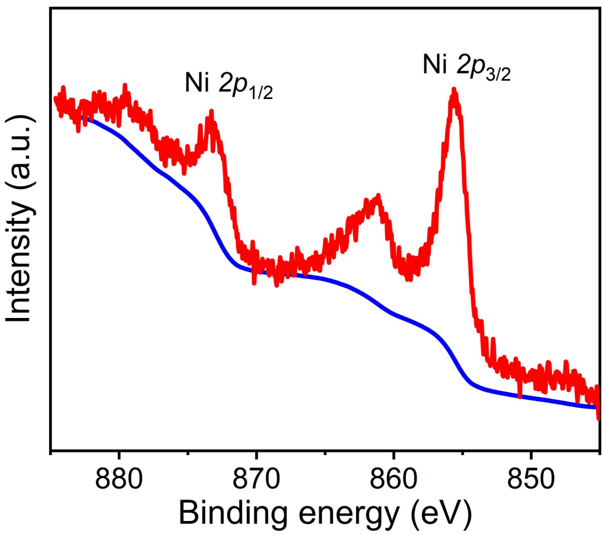

Supplementary Figure 11 | XPS spectrum of Ni *2p* for NiOOH/α-Fe_2O_3. The Ni $2p_{3/2}$ binding energy at 855.6 eV and Ni $2p_{1/2}$ binding energy at 873.0 eV are assigned to Ni^{III} state from NiOOH [18].

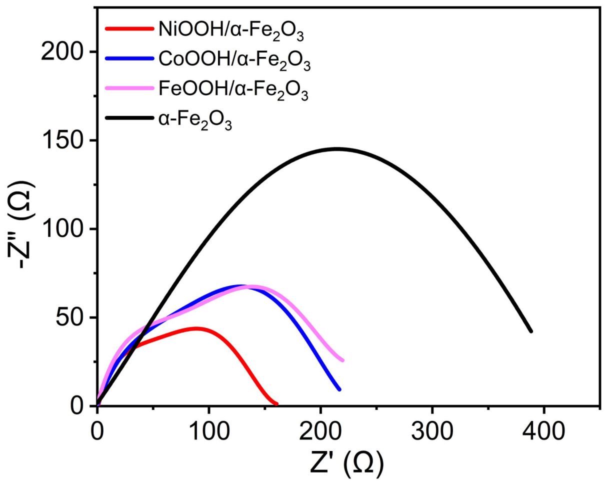

Supplementary Figure 12 | EIS Nyquist plots of α-Fe_2O_3, FeOOH/α-Fe_2O_3, CoOOH/α-Fe_2O_3, and NiOOH/α-Fe_2O_3 measured at 1 V under AM 1.5G one-sun illumination in Ar-purged 1 M aqueous solution of KOH (pH~13.6) with 10 mM glucose.

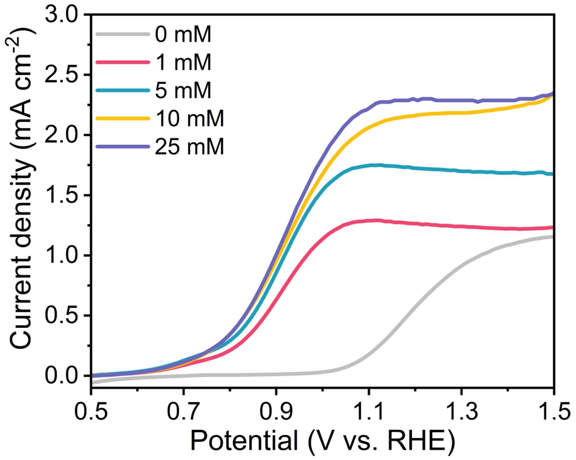

Supplementary Figure 13 | LSV curves of GOR over NiOOH/α-Fe_2O_3 with different glucose concentrations under AM 1.5G one-sun illumination.

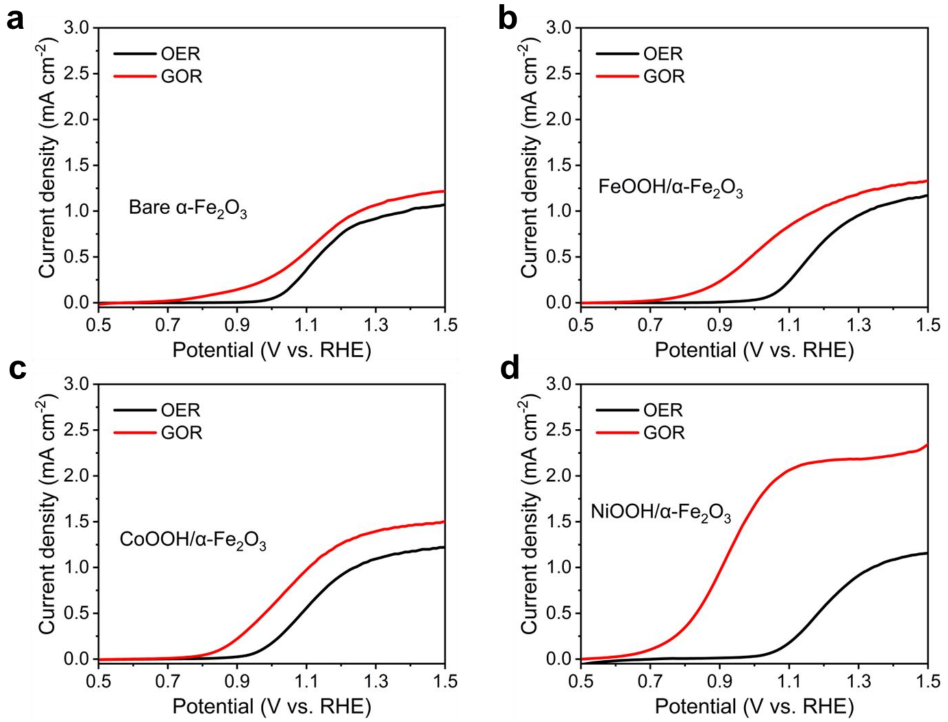

Supplementary Figure 14 | LSV curves for OER and GOR over (a) bare α-Fe_2O_3, (b) FeOOH/α-Fe_2O_3, (c) CoOOH/α-Fe_2O_3, and (d) NiOOH/α-Fe_2O_3 under AM 1.5G one-sun illumination. The onset potential of OER was observed at around 1 V for all the samples. Among all the samples, NiOOH/α-Fe_2O_3 exhibited the best performance towards GOR in terms of onset potential and photocurrent density.

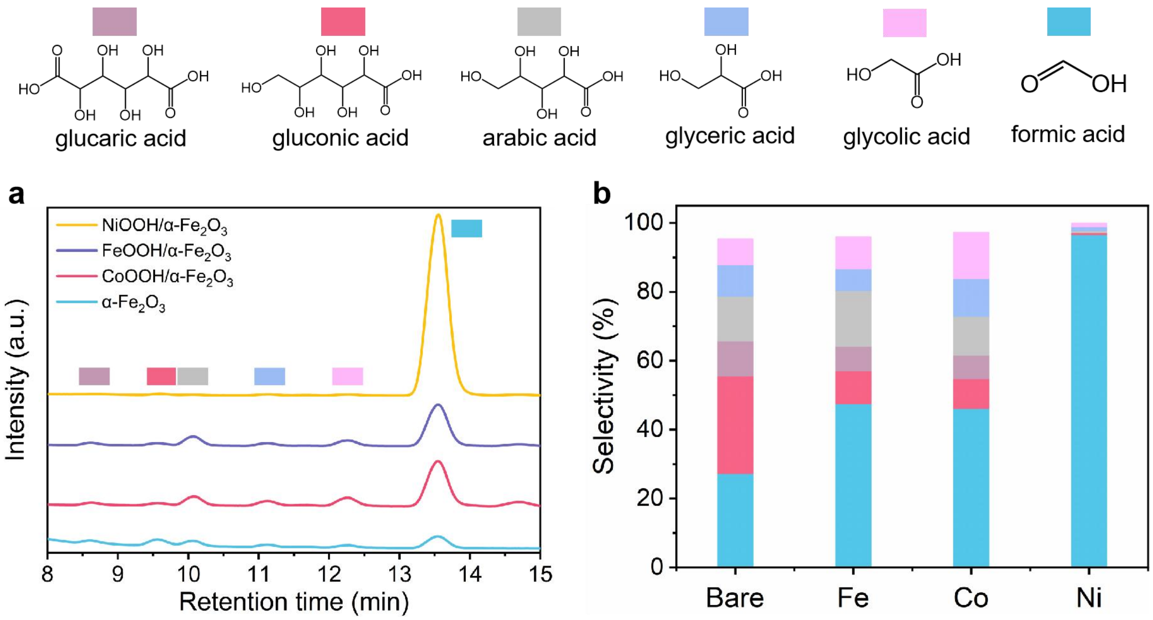

Supplementary Figure 15 | Product analysis of electrolyte after 2 h GOR at 1 V under AM 1.5G one-sun illumination. (a) HPLC chromatograms and (b) product selectivity. NiOOH/α-Fe_2O_3 exhibited the highest formate selectivity. Other products detected in the electrolyte were glucaric acid ($C_6H_{10}O_8$), gluconic acid ($C_6H_{12}O_7$), arabic acid ($C_5H_{10}O_6$), glyceric acid ($C_3H_6O_4$) and glycolic acid ($C_2H_4O_3$). Erythric acid ($C_4H_8O_5$) may also exist in the products because its retention time in HPLC chromatogram is very close to that of arabic acid ($C_5H_{10}O_6$). The total carbon balance is over 95% for all the samples.

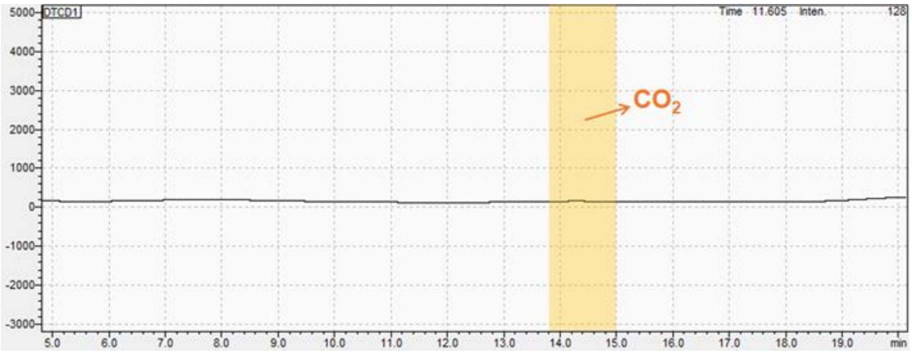

Supplementary Figure 16 | Gas chromatography spectrum of products after 2 h GOR at 1 V under AM 1.5G one-sun illumination over NiOOH/α-Fe_2O_3.

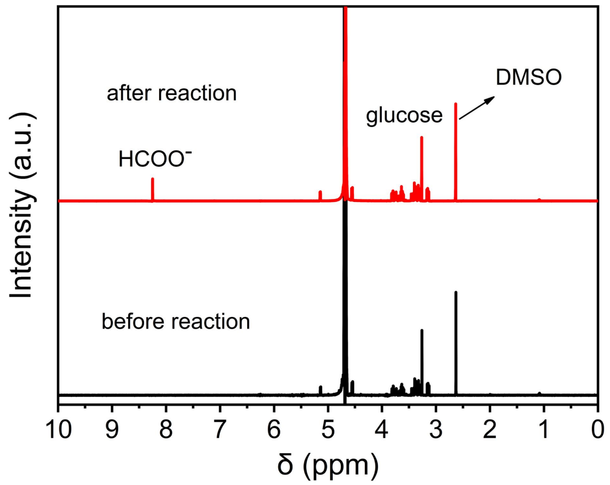

Supplementary Figure 17 | ^{1}H NMR spectra of electrolyte after 2 h GOR at 1 V under AM 1.5G one-sun illumination over NiOOH/α-Fe_2O_3 photoanode. A sample before the PEC reaction is shown for comparison. DMSO was used as the internal standard. The evolution of formate signal at 8.2 ppm was observed after the PEC reaction.

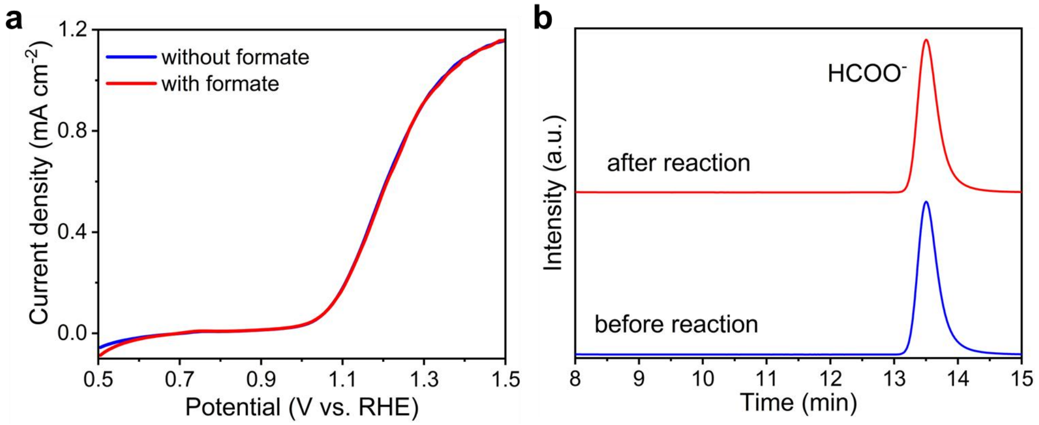

Supplementary Figure 18 | a LSV curves over NiOOH/α-Fe_2O_3 in Ar-purged 1 M aqueous solution of KOH with and without formate added under AM 1.5G one-sun illumination. **b** HPLC chromatograms of electrolytes before and after 2 h GOR at 1 V under AM 1.5G one-sun illumination over NiOOH/α-Fe_2O_3. It shows that formate is stable in the PEC reaction system.

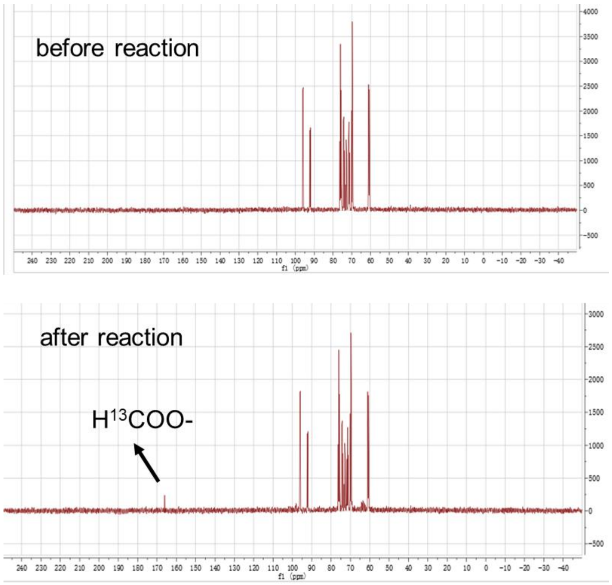

Supplementary Figure 19 | ^{13}C NMR spectra of electrolyte before and after the PEC reaction using ^{13}C-labeled glucose as the reactant substrate. The experiment was conducted at 1 V for 2 h in Ar-purged 1 M aqueous solution of KOH (pH~13.6) with 10 mM ^{13}C-glucose under AM 1.5G one-sun illumination.

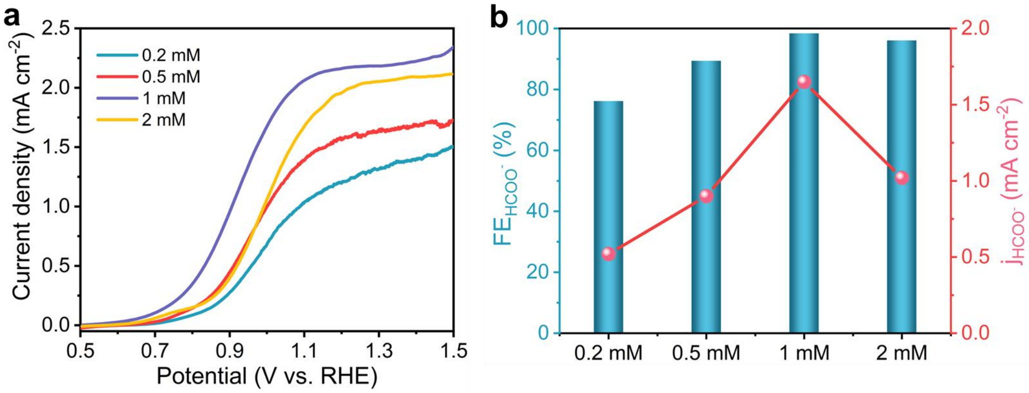

Supplementary Figure 20 | Influence of $Ni(NO_3)_2$ concentration in the preparation of NiOOH/α-Fe_2O_3 on the PEC GOR performance. **a** LSV curves. **b** FEs and partial current density of formate (j_{HCOO^-}). All the experiments were conducted at 1 V for 2 h in Ar-purged 1 M aqueous solution of KOH (pH~13.6) with 10 mM glucose under AM 1.5G one-sun illumination.

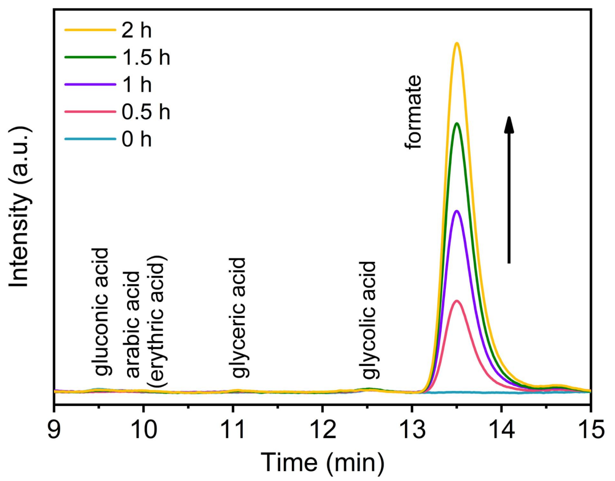

Supplementary Figure 21 | HPLC chromatograms of electrolyte as a function of PEC reaction time. The experiment was conducted at 1 V in Ar-purged 1 M aqueous solution of KOH (pH~13.6) with 10 mM glucose under AM 1.5G one-sun illumination.

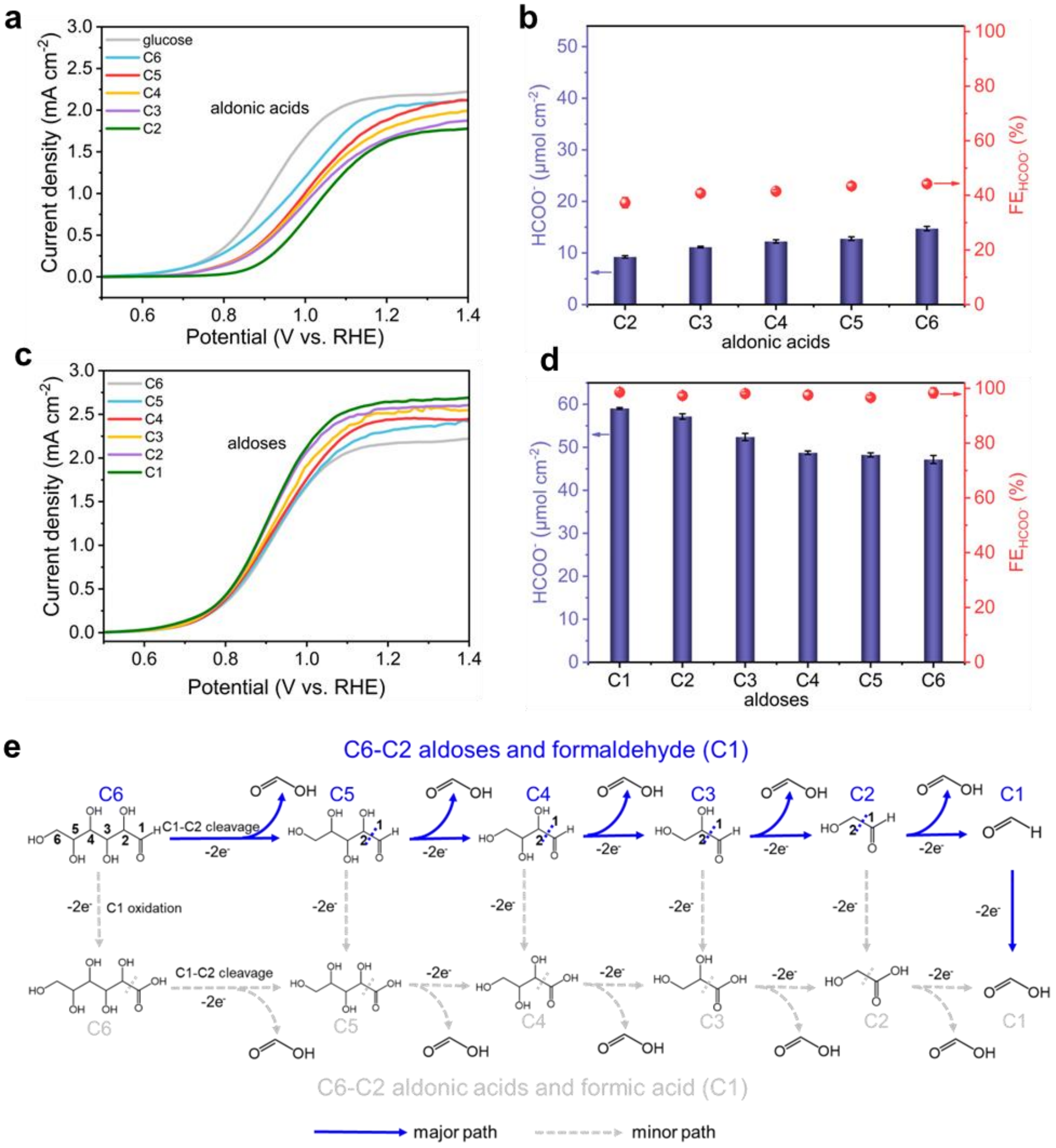

Supplementary Figure 22 | a LSV curves, **b** formate productivity and FEs using 10 mM aldonic acids as the reactant substrates. **c** LSV curves, **d** formate productivity and FEs using 10 mM aldoses as the reactant substrates. **e** Proposed reaction pathway of PEC glucose oxidation to formate. All the experiments were conducted at 1 V for 2 h in Ar-purged 1 M aqueous solution of KOH (pH~13.6) under AM 1.5G one-sun illumination. The error bars represent one standard deviation of three independent measurements.

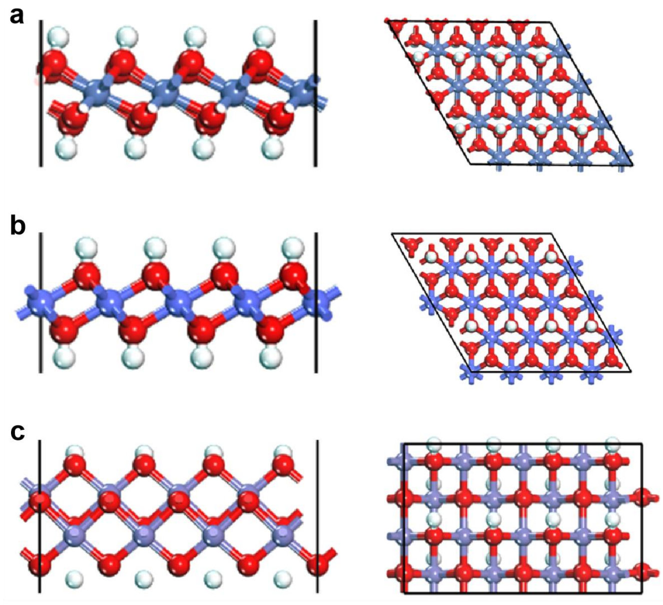

Supplementary Figure 23 | Side and top view of atomic models of (a) NiOOH, (b) CoOOH and (c) FeOOH.

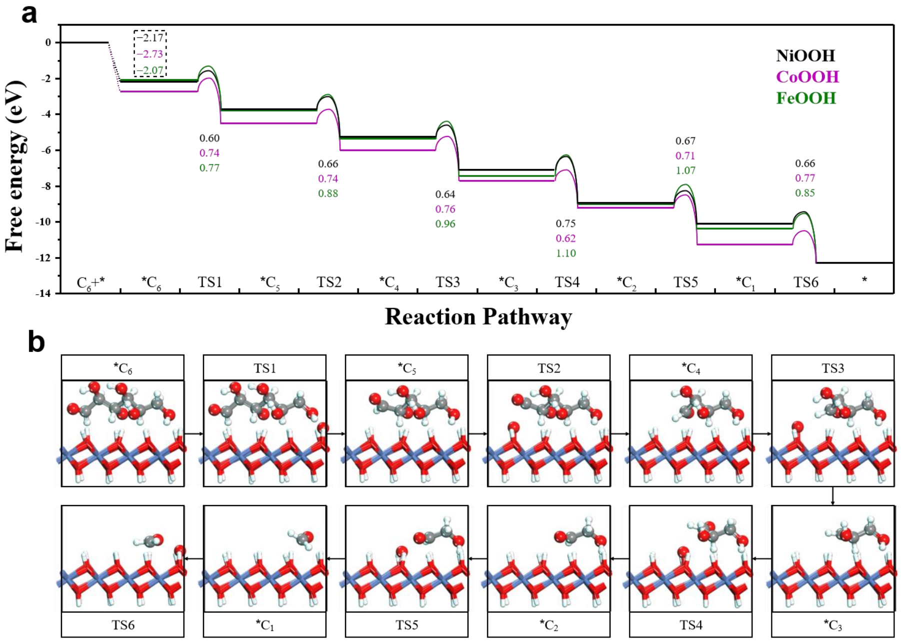

Supplementary Figure 24 | Theoretical calculations of glucose conversion on NiOOH, CoOOH and FeOOH. (a) Gibbs free energy profiles. (b) The corresponding intermediate configurations during the process of glucose conversion.

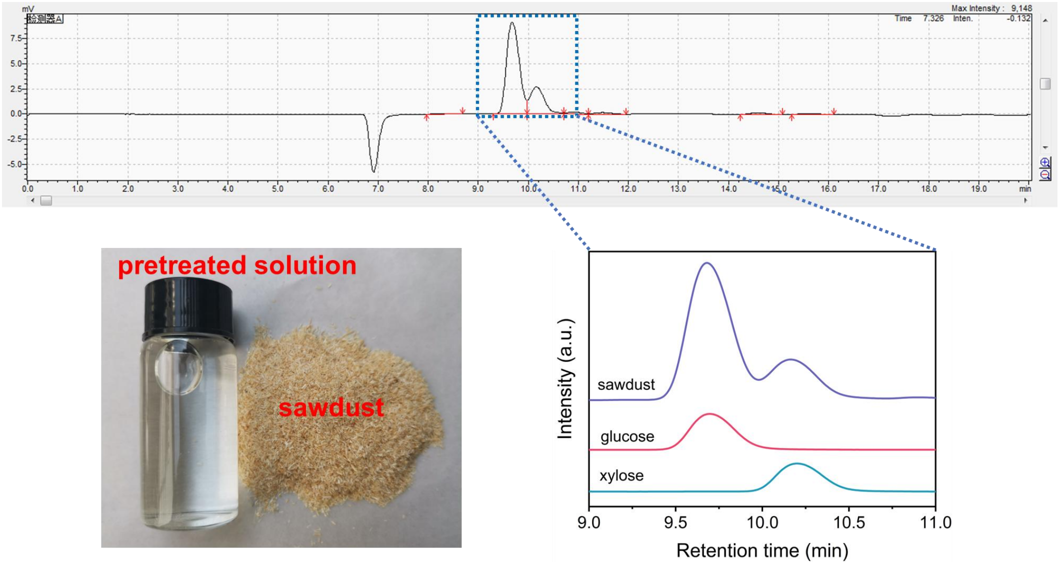

Supplementary Figure 25 | Representative HPLC spectrum of sugar solution after acidic pretreatment of raw biomass. Here exemplified by use the poplar sawdust as the substrate. It can be detected that the sugar solution mainly contains glucose and xylose. A small amount of cellobiose was also detected at the retention time of 8.4 min.

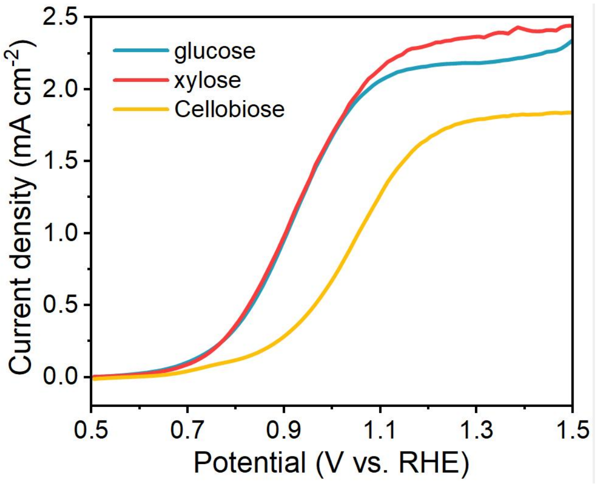

Supplementary Figure 26 | LSV curves over NiOOH/α-Fe_2O_3 using 10 mM glucose, xylose and cellobiose as the reactant substrates. All the experiments were conducted in Ar-purged 1 M aqueous solution of KOH (pH~13.6) under AM 1.5G one-sun illumination.

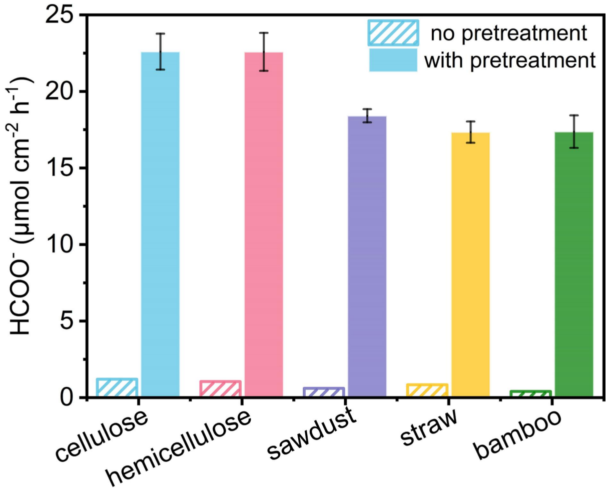

Supplementary Figure 27 | The effect of pretreatment on formate productivity. All the experiments were conducted at 1 V for 2 h over NiOOH/α-Fe_2O_3 in Ar-purged 1 M aqueous solution of KOH (pH~13.6) under AM 1.5G one-sun illumination. The error bars represent one standard deviation of two independent measurements.

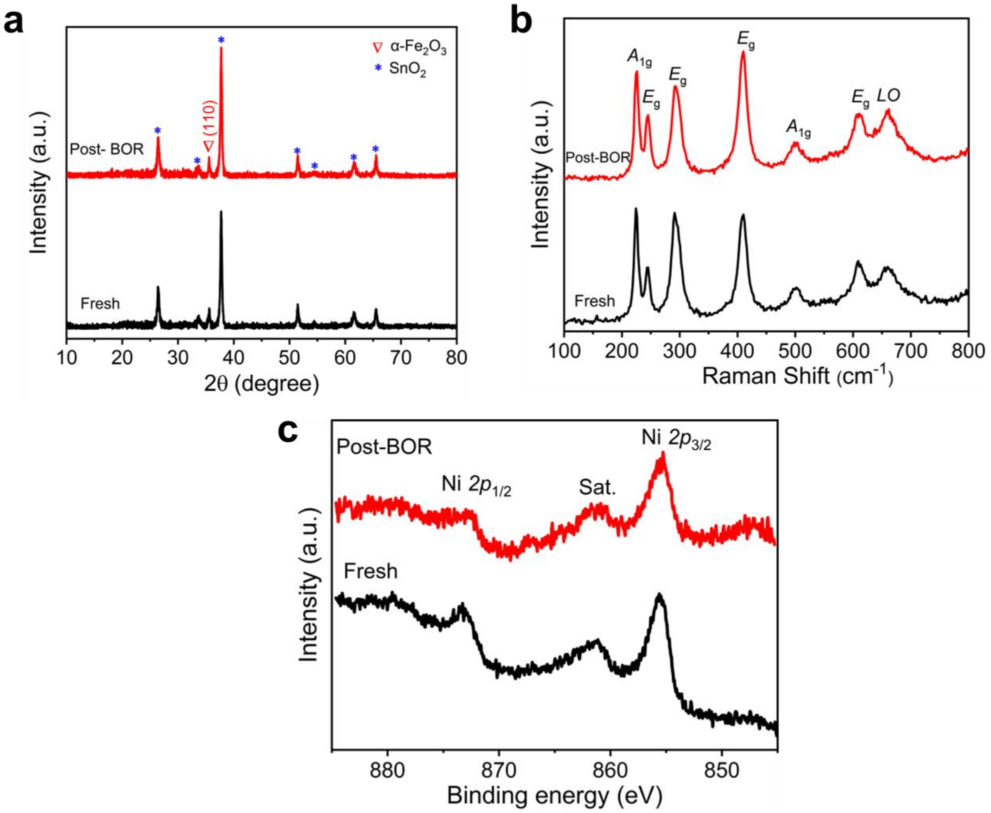

Supplementary Figure 28 | a XRD patterns, **b** Raman spectra, **c** XPS spectra of Ni *2p* of NiOOH/α-Fe_2O_3 before reaction (black line) and after 100 h PEC BOR reaction (red line).

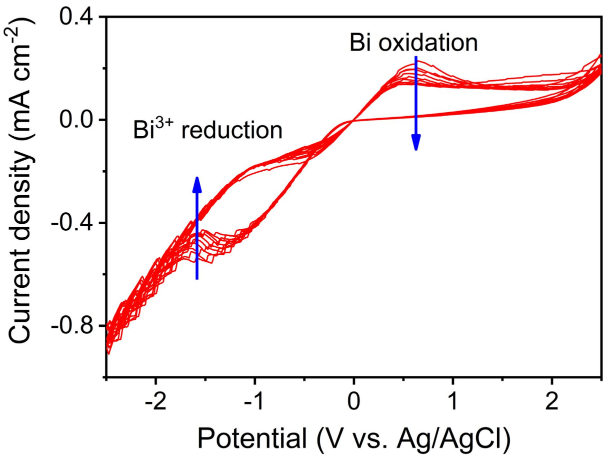

Supplementary Figure 29 | Cyclic voltammetry curves of Bi cocatalyst deposition on GaN/Si from −2.5 V to +2.5 V vs. Ag/AgCl.

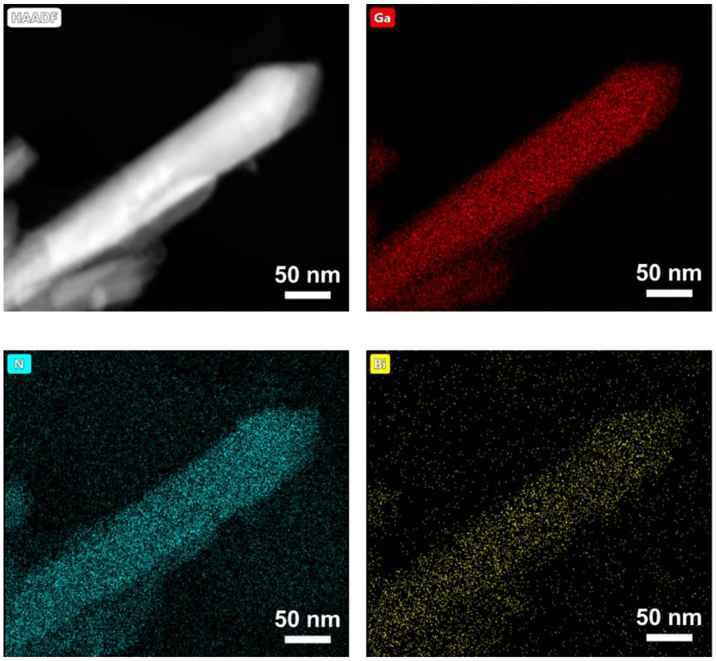

Supplementary Figure 30 | STEM-EDX mapping of Bi decorated GaN nanowires.

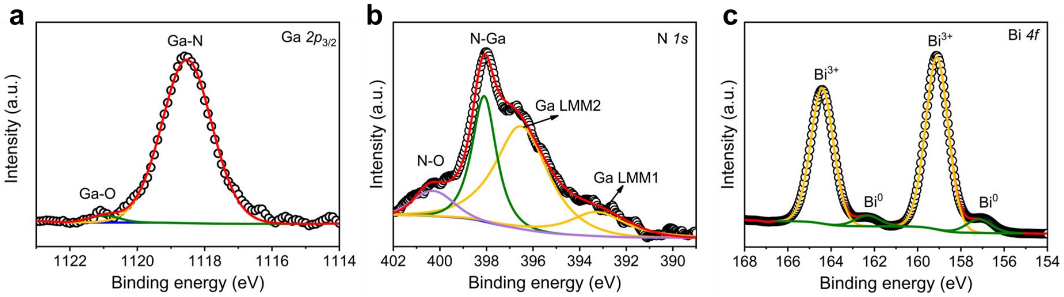

Supplementary Figure 31 | XPS spectra of **a** Ga *2p*$_{3/2}$, **b** N *1s*, and **c** Bi *4f* of Bi/GaN/Si.

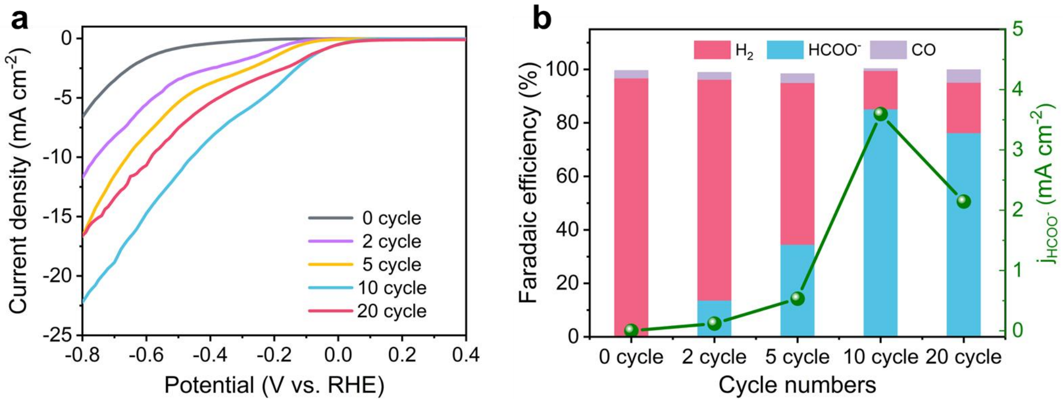

Supplementary Figure 32 | PEC performance of Bi/GaN/Si photocathodes with varied electrodeposition cycles of Bi. **a** LSV curves. **b** FEs of different products and j_{HCOO^-}. The electrodeposition was realized by sweeping potential between −2.5 to +2.5 V with different cyclic voltammetry numbers.

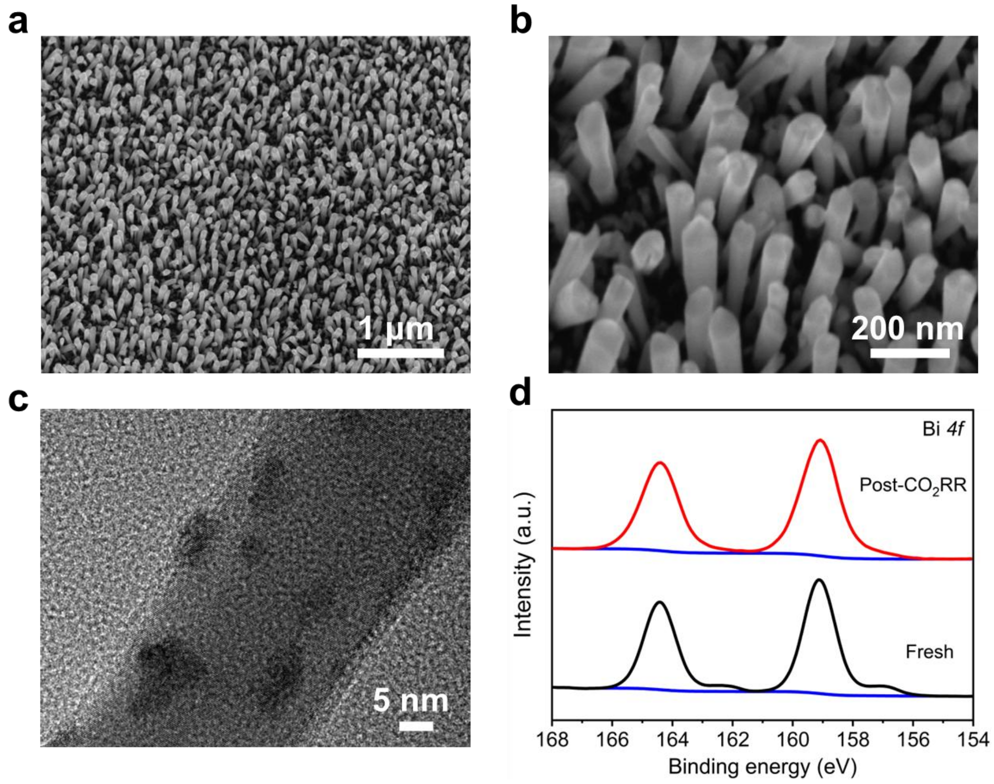

Supplementary Figure 33 | Bi/GaN/Si photocathode characterization after PEC CO_2RR reaction. **a,b** SEM images. **c** TEM image. **d** XPS spectra of Bi *4f*.

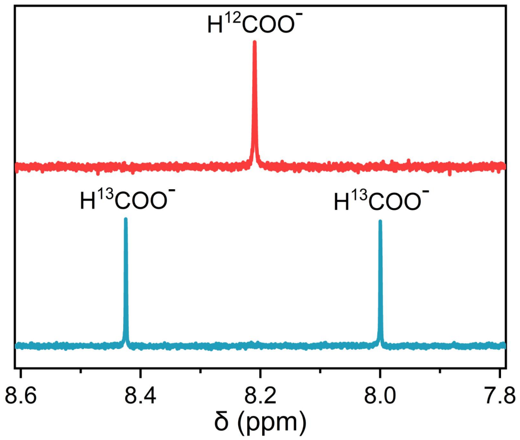

Supplementary Figure 34 | ^{1}H NMR analysis of the products of ^{13}C isotopic CO_2RR experiment. The ^{13}C isotopic experiment was conducted in $^{13}CO_2$-saturated 0.5 M $KH^{13}CO_3$ aqueous solution.

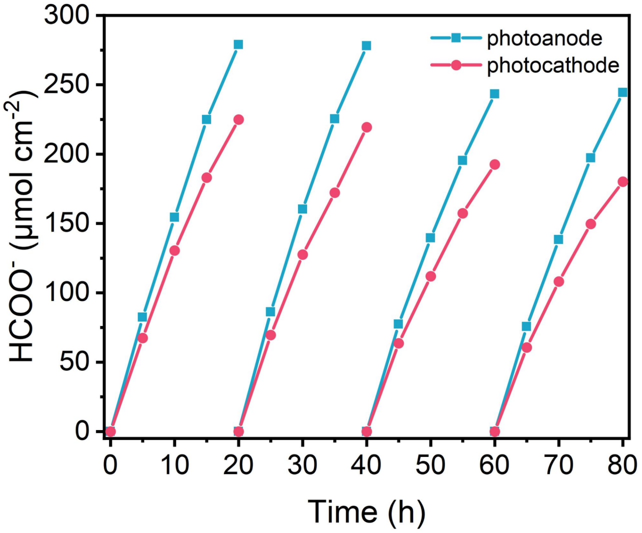

Supplementary Figure 35 | Amount of formate produced at photoanode and photocathode in 80 h test.

Supplementary Table 1. Compositional analysis of raw biomass substrates and the corresponding sugars solutions after pretreatment.

Raw biomass	Before pretreatment[a]			After pretreatment[b]		(Hemi)cellulose-to-glucose/xylose yield (%)
	Cellulose (wt%)	Hemicellulose (wt%)	Lignin (wt%)	Glucose (g)	Xylose (g)	
Sawdust	58.12	19.00	16.53	0.193	0.051	63.28
Straw	40.02	24.93	4.81	0.145	0.067	65.28
Bamboo	49.13	21.58	20.05	0.163	0.066	64.77

[a] The Van Soest method[19,20] was used to quantify the component of cellulose, hemicellulose and lignin in biomass.

[b] The pretreatment was conducted by refluxing in H_2SO_4 solution at 100 °C for 5 h. Raw biomass feedstock (0.5 g), solution (100 ml).

Supplementary Table 2. Performance comparison of representative Si-based photocathodes for PEC CO_2 reduction into $HCOO^-$.

Photocathode	FE_{HCOO^-}	Overpotential[a] (V)	Refs
Bi/GaN/Si	85.2% at −0.2 V vs. RHE	0	this work
Sn-pNWs/n^+p-Si	59.2% at −0.4 V vs. RHE	0.2	21
NPhN-Ru$(CP)_2^{2+}$-RuCt/NiO/GaN NWs/Si	69% at −0.25 V vs. RHE	0.05	22
CuS NPs/GaN NWs/p-n^+ Si	70.2% at −1.0 V vs. RHE	0.8	23
Sn NP/GaN NW/Si	76.9% at −0.53 V vs. RHE	0.33	24
Sn NPs/p-Si NWs	88% at −0.876 V vs. RHE	0.676	25
Bi/p-Si	94% at −0.32 V vs. RHE	0.12	26

[a] Overpotential is referred to the equilibrium potential of CO_2/HCOOH at −0.2 V vs. RHE.

Supplementary Table 3. Performance comparison of representative unbiased PEC $HCOO^-$ production from CO_2 and H_2O.

Material	$HCOO^-$ productivity ($\mu mol\ cm^{-2}\ h^{-1}$)	Duration time (h)	Refs
FeOOH/$BiVO_4$//ClFDH/TiO_2/CFO	0.098	12	27
InP/[MCE2-A+MCE4]//TiO_2/Pt	0.22	24	28
FeOOH/$BiVO_4$//perovskite//3D TiN-ClFDH	1.06	8	29
$SrTiO_3$:La,Rh//Au//RuO_2-$BiVO_4$:Mo	1.12	34	30
Ru(MeCN)CO_2C_3Py-P/TiO_2/N,Zn-Fe_2O_3 /Cr_2O_3//$SrTiO_{3-x}$	1.55	3	31
r-STO//lnP/[RuCP]	1.93	3	32
$CuFeO_2$/CuO//Pt	2.5	24	33
$BiVO_4$//PVK/IO-TiO_2/FDH	7	10	34
NiOOH/Fe_2O_3//Si PV//Bi/GaN/Si	23.27	80	this work

Supplementary Note 1 Calculation of solar to chemical conversion efficiency

Due to the Faradaic efficiencies of formate production were different at anode and cathode, formate were produced based on two overall reactions as shown below. In both reactions, the anode reaction was glucose oxidation to formate. However, the cathode reactions were either CO_2 reduction to formate or H_2O reduction to H_2.

(Standard molar free energy of formation $\triangle G_f$): $C_6H_{12}O_6$ (s): −910.56 kJ/mol; CO_2 (g): −394.4 kJ/mol; H_2O (l): −237.13 kJ/mol; HCOOH (l): −361.3 kJ/mol; H_2 (g):0 kJ/mol.

Overall reaction 1 (R1): 1/12 $C_6H_{12}O_6$ + 1/2 CO_2 + 1/2 H_2O → HCOOH $\triangle G_{R1}$=30.345 kJ/mol

Overall reaction 2 (R2): 1/6 $C_6H_{12}O_6$ + H_2O → HCOOH + H_2 $\triangle G_{R2}$=27.59 kJ/mol

The solar to chemical conversion efficiency (η) was calculated according to the following equation:

$$\eta = \frac{N_{formate}^{R1} \times \Delta G_{R1} + N_{formate}^{R2} \times \Delta G_{R2}}{P_{solar} \times t}$$

where $N_{formate}^{R1}$ and $N_{formate}^{R2}$ are formate production amount via reaction 1 and 2, respectively, P_{solar} is the input solar power, and t is the reaction time. The total amount of formate produced at the anode and cathode sides after 80 h test were detected to be 1044 μmol cm^{-2} and 817 μmol cm^{-2}, respectively (Figure S35). Twice the amount of formate produced at the cathode can be regarded as formate produced via R1 reaction (1634 μmol cm^{-2}). Besides, formate produced at anode side is higher than that of cathode side and the extra formate at anode was regarded as produced via R2 reaction (227 μmol cm^{-2}). Thus, η was calculated to be ~0.19%.

$$\eta=(1634\times30.345+227\times27.59)/(100\times80\times3600)=0.194\%$$

References

1 Peerakiatkhajohn, P. *et al.* Stable hematite nanosheet photoanodes for enhanced photoelectrochemical water splitting. *Adv. Mater.* **28**, 6405–6410 (2016).

2 Kay, A., Cesar, I. & Grätzel, M. New benchmark for water photooxidation by nanostructured α-Fe_2O_3 films. *J. Am. Chem. Soc.* **128**, 15714–15721 (2006).

3 Kment, S. *et al.* Photoanodes with fully controllable texture: the enhanced water splitting efficiency of thin hematite films exhibiting solely (110) crystal orientation. *ACS Nano* **9**, 7113–7123 (2015).

4 Zhang, H. M. *et al.* Gradient tantalum-doped hematite homojunction photoanode improves both photocurrents and turn-on voltage for solar water splitting. *Nat. Commun.* **11**, 4622 (2020).

5 Spray, R. L. & Choi, K. S. Photoactivity of transparent nanocrystalline Fe_2O_3 electrodes prepared via anodic electrodeposition. *Chem. Mater.* **21**, 3701–3709 (2009).

6 Zeng, Q. Y. *et al.* A novel in situ preparation method for nanostructured α-Fe_2O_3 films from electrodeposited Fe films for efficient photoelectrocatalytic water splitting and the degradation of organic pollutants. *J. Mater. Chem. A* **3**, 4345–4353 (2015).

7 Zhang, N. S. *et al.* Paving the road toward the use of β-Fe_2O_3 in solar water splitting: Raman identification, phase transformation and strategies for phase stabilization. *Natl. Sci. Rev.* **7**, 1059–1067 (2020).

8 Jang, J.-W. *et al.* Enabling unassisted solar water splitting by iron oxide and silicon. *Nat. Communs.* **6**, 7447 (2015).

9 Chen, Y. B. *et al.* Modification of Ti-doped hematite nanowires with a NiOx buffer layer for improved photoelectrochemical performance. *Appl. Phys. Lett.* **119**, 083901 (2021).

10 Peerakiatkhajohn, P. *et al.* Stable hematite nanosheet photoanodes for enhanced photoelectrochemical water splitting. *Adv. Mater.* **28**, 6405–6410 (2016).

11 Xu, Z. *et al.* Interface manipulation to improve plasmon-coupled photoelectrochemical water splitting on α-Fe_2O_3 photoanodes. *Chemsuschem* **11**, 237–244 (2018).

12 Zhao, Y. K. *et al.* α-Fe_2O_3 as a versatile and efficient oxygen atom transfer catalyst in combination with H_2O as the oxygen source. *Nat. Catal.* **4**, 684–691 (2021).

13 Liu, P. F. *et al.* Ultrathin hematite photoanode with gradient Ti doping. *Research* **2020**, 5473217 (2020).

14 Wu, Y. H. *et al.* Combinatorial studies on wet-chemical synthesized Ti-doped α-Fe_2O_3: how does Ti^{4+} improve photoelectrochemical activity? *ACS Appl. Nano Mater.* **1**, 3145–3154 (2018).

15 Zandi, O., Klahr, B. M. & Hamann, T. W. Highly photoactive Ti-doped α-Fe_2O_3 thin film electrodes: resurrection of the dead layer. *Energ Environ. Sci.* **6**, 634–642 (2013).

16 Hill, J. C., Landers, A. T. & Switzer, J. A. An electrodeposited inhomogeneous metal–insulator–semiconductor junction for efficient photoelectrochemical water oxidation. *Nat. Mater.* **14**, 1150–1155 (2015).

17 Pei, L. *et al.* Mg-doped Ta_3N_5 nanorods coated with a conformal CoOOH layer for water

oxidation: bulk and surface dual modification of photoanodes. *J. Mater. Chem. A* **5**, 20439–20447 (2017).

18 Biesinger, M. C., Lau, L. W. M., Gerson, A. R. & Smart, R. S. C. The role of the auger parameter in XPS studies of nickel metal, halides and oxides. *Phys. Chem. Chem. Phys.* **14**, 2434–2442 (2012).

19 Vansoest, P. J. Use of detergents in analysis of fibrous feeds II a rapid method for determination of fiber and lignin. *J. Assoc. Off. Anal. Chem.* **46**, 829–835 (1963).

20 Vansoest, P. J. & Wine, R. H. Use of detergents in analysis of fibrous feeds IV determination of plant cell-wall constituents. *J. Assoc. Off. Anal. Chem.* **50**, 50–55 (1967).

21 Rao, K. R. *et al.* Photoelectrochemical reduction of CO_2 to HCOOH on silicon photocathodes with reduced SnO_2 porous nanowire catalysts. *J. Mater. Chem. A* **6**, 1736–1742 (2018).

22 Shan, B. *et al.* Binary molecular-semiconductor p–n junctions for photoelectrocatalytic CO_2 reduction. *Nat. Energy* **4**, 290–299 (2019).

23 Dong, W. J. *et al.* CuS-decorated GaN nanowires on silicon photocathodes for converting CO_2 mixture gas to HCOOH. *J. Am. Chem. Soc.* **143**, 10099–10107 (2021).

24 Zhou, B. *et al.* A GaN:Sn nanoarchitecture integrated on a silicon platform for converting CO_2 to HCOOH by photoelectrocatalysis. *Energy Environ. Sci.* **12**, 2842–2848 (2019).

25 Choi, S. K. *et al.* Sn-coupled p-Si nanowire arrays for solar formate production from CO_2. *Adv. Energy Mater.* **4**, 1301614 (2014).

26 Ding, P. *et al.* Controlled chemical etching leads to efficient silicon–bismuth interface for photoelectrochemical CO_2 reduction to formate. *Mater. Today Chem.* **11**, 80–85 (2019).

27 Kuk, S. K. *et al.* CO_2-reductive, copper oxide-based photobiocathode for Z-scheme semi-artificial leaf structure. ChemSusChem **13**, 2940–2944 (2020).

28 Sato, S. *et al.* Selective CO_2 conversion to formate conjugated with H_2O oxidation utilizing semiconductor/complex hybrid photocatalysts. *J. Am. Chem. Soc.* **133**, 15240–15243 (2011).

29 Kuk, S. K. *et al.* Continuous 3D titanium nitride nanoshell structure for solar-driven unbiased biocatalytic CO_2 reduction. *Adv. Energy Mater.* **9**, 1900029 (2019).

30 Wang, Q. *et al.* Molecularly engineered photocatalyst sheet for scalable solar formate production from carbon dioxide and water. *Nat. Energy* **5**, 703–710 (2020).

31 Sekizawa, K., Sato, S., Arai, T. & Morikawa, T. Solar-driven photocatalytic CO_2 reduction in water utilizing a ruthenium complex catalyst on p-type Fe_2O_3 with a multiheterojunction. *ACS Catal.* **8**, 1405–1416 (2018).

32 Arai, T., Sato, S., Kajino, T. & Morikawa, T. Solar CO_2 reduction using H_2O by a semiconductor/metal-complex hybrid photocatalyst: enhanced efficiency and demonstration of a wireless system using $SrTiO_3$ photoanodes. *Energy Environ. Sci.* **6**, 1274–1282 (2013).

33 Kang, U. *et al.* Photosynthesis of formate from CO_2 and water at 1% energy efficiency via copper iron oxide catalysis. *Energy Environ. Sci.* **8**, 2638–2643 (2015).

34 Edwardes Moore, E. *et al.* A semi-artificial photoelectrochemical tandem leaf with a

CO_2-to-formate efficiency approaching 1 %. *Angew. Chem. Int. Ed.* **60**, 26303–26307 (2021).
